# The African Genomic Medicine Training Initiative (AGMT): Showcasing a Community and Framework Driven Genomic Medicine Training for Nurses in Africa

**DOI:** 10.3389/fgene.2019.01209

**Published:** 2019-12-20

**Authors:** Victoria Nembaware, African Genomic Medicine Training Initiative, Omar Abidi, Nicola Mulder

**Affiliations:** ^1^Division of Human Genetics, Faculty of Health Sciences, University of Cape Town, Cape Town, South Africa; ^2^Computational Biology Division, Department of Integrative Biomedical Sciences, IDM, Faculty of Health Sciences, University of Cape Town, Cape Town, South Africa

**Keywords:** genomic medicine, Africa, precision medicine, training, nurses, competencies, Kern's six step model

## Abstract

The potential of genomic medicine in improving the quality of healthcare both at population and individual-level is well-recognized globally. However, successful adoption of genetic and genomic evidence into clinical practice depends on training the healthcare workforce and clinical researchers in genomic medicine. Due to limited expertise in the medical genetics and genomics field, widespread uptake largely depends on task-shifting for the implementation of genomic medicine implementation to key healthcare professionals such as nurses. Their knowledge would be developed through courses aimed at professional development. Globally, trainers, and training initiatives in genomic medicine are in early stages of development, but resource limited settings such as the African continent face additional logistical and institutional challenges. The African Genomic Medicine Training (AGMT) Initiative was conceived during a combined conference of the African Society of Human Genetics (AfSHG) and the Human Heredity and Health in Africa Consortium (H3Africa) in 2016, Senegal, in response to the needs for developing knowledge and skills in genomic medicine. AGMT was established to implement a sustainable genomic medicine training initiative primarily for healthcare professionals who are not geneticists but are nurses, doctors, and pharmacists in Africa. This paper reports on the establishment of the AGMT initiative and the strategies developed and piloted by this initiative in designing and implementing an accredited frame-work and community-based blended learning course for nurses across 11 African countries. The global implementation experiences, outcomes and lessons learnt are highlighted. The AGMT initiative strategy takes advantage of existing research consortia and networks to train and create a pool of trainers and has adopted evidence-based approaches to guide curriculum and content development/adaptation. This initiative established the first Africa-wide online blended learning genomic medicine course which forms the basis from which to develop courses for other healthcare professionals and the wider public.

## Background

Historically, knowledge translation of genomic knowledge for healthcare in Africa has been challenged by the dearth of genomic data from people of recent African origin (Popejoy and Fullerton, 2016). However, recent initiatives such as the Human, Heredity, and Health in Africa Consortium (H3Africa) (Dandara et al., 2014a; Rotimi et al., 2014), H3ABioNet (Mulder et al., 2016), and MalariaGen (Achidi et al., 2008) aim to build capacity for genomics research in Africa, and are challenging the existing norms (Gurdasani et al., 2015). Cumulating results from genomic projects of human health and disease projects are helping explain African-specific susceptibility and variability in disease severity to conditions such as kidney-related diseases (Cooper et al., 2017) and sickle cell disease (SCD) (Pule et al., 2015). The application of genomics information in optimizing treatment has given rise to development of pharmacogenomics-based dosing of antiretroviral therapy (ART) (Dandara et al., 2014b; Skelton et al., 2014). Large-scale genomic characterization of African populations holds great promise for identification of additional health-linked genetic variants relevant to the understanding of possible genomic drivers of the high burden of infectious diseases and the growing prevalence of noncommunicable diseases in Africa (Wonkam and Mayosi, 2014; Tekola-Ayele and Rotimi, 2015). Therefore, in anticipation of the changing African genomic landscape, there is an urgent need for strategies to translate this genomic knowledge into clinical practice and augment clinical decisions.

Efforts to translate genomics into clinical practice face a number of barriers which include limited resources to sequence and characterize human genomes from African populations. In addition, there is lack of access to next generation technologies and analytical capabilities in Africa and, policies that are silent on genetics and genomics and medical curricula that are not adequate in teaching genetics/genomics concepts, leading to limited appreciation of the utility of genomic knowledge in healthcare (Wonkam et al., 2006; Muzoriana et al., 2017). In addition, African countries lack the critical mass of experts in genetics, genomics, data science, and bioinformatics required to implement country-specific genomic medicine driven healthcare. For example, South Africa is the only African country with a critical mass of skilled genetic counsellors and medical geneticists, key personnel for the implementation of genomic medicine (Abacan et al., 2019).Therefore, cost-effective strategies are urgently required to promote incorporation of genomics research and findings in healthcare in Africa for quality health outcomes. Most of the developed world is moving to adopting genomics in its health care programs, however, Africa is still lagging behind, a trend that will continue to widen existing health disparities between developed and developing countries.

Globally, several introductory genomic medicine courses have been developed and implemented, tailored for specific healthcare workers such as nurses (Nembaware et al., 2016), and other healthcare professionals (https://www.genomicseducation.hee.nhs.uk). However, several of the genomic medicine curricula are characterized by numerous shortcomings, which include limited development of competencies in a systematic manner. Mapping and alignment of curricula and competencies are slowly being integrated into genomics curricula development (Jenkins et al., 2015). Competencies in genomics and genetics for nurses are publicly available online (Jenkins and Calzone, 2007; Kirk et al., 2014), a noteworthy competency resource was developed by the Inter-Society Coordinating Committee for Physician Education in Genomics (ISCC) based on five "Entrustable Professional Activities" EPAs (Korf et al., 2014). Another short-coming of most existing genomic medicine curricula is the limited application of well-established curriculum development frameworks such as the Kern's six step model (Kern and Thomas, 2009). This model is a widely used systematic curriculum development approach which links curricula to healthcare needs and promotes continual curriculum monitoring and evaluation (Khamis et al., 2016). This model has the added advantage of being adaptable to suit the needs of the implementers (Khamis et al., 2016).

The existing publicly available genomic medicine curricula require tailoring of competencies and content for the African context due to the continent's diverse cultures, disease burdens, and healthcare facilities and resources. In addition, reported challenges from an informal online survey in training genetics and genomics highlight lack of expertise, and lack of resources and funds (https://training.h3abionet.org/AGMC_2016/outputs/). To address the highlighted training needs and establish a foundation for genomic medicine in the region, the African Genomic Medicine Training Initiative (AGMT) was initiated to pool expertise and resources from across Africa to develop a training program for African healthcare professionals, which could be further tailored across the diverse countries. The goals of the AGMT initiative are to:

- establish a comprehensive, adaptable and coordinated genomic medicine curriculum and training plan for Africa;
- develop distributed model/flagship training programs based on the curricula;
- establish genomic medicine critical quality indicators to assess competency levels of healthcare professionals in Africa; and
- establish a monitoring and evaluation system to capture the rate of adoption of the curriculum once developed and to track trainees

This article focuses only on the curriculum development objective of the AGMT and outlines the steps taken in the development and implementation of the genomic medicine curriculum, firstly targeted at nurses in Africa. The article demonstrates how the AGMT initiative adapted the Kern's six-step model for the development of a medical curriculum. In addition, the Kern's six-step model was modified to incorporate a competency mapping approach developed by the International Society of Computational Bioinformatics (ISCB) (Mulder et al., 2018). Formal medical educational programs have aims and goals that are often not clearly articulated and, in some instances, poorly understood by key constituents inside and outside of the formal education system (Kern and Thomas, 2009). Using a model/framework to develop the curriculum helps clarify aims and objectives around which the curriculum is structured (Kern and Thomas, 2009). The curriculum becomes the official documentation that includes the goals of teaching and learning; the instructional methods and materials as well as the assessment. The curriculum reflects the envisaged aspirations of society as well as the curriculum that is ultimately implemented. This helps the newly trained medical educators meet the needs of their students, patients, and other key stakeholders. The use of a framework to guide the development of genomic medicine training for Africa also presents an opportunity to implement formal evaluations and studies to share lessons and to learn from.

## Description

A workshop aimed at establishing AGMT initiative was conducted during a combined conference of the African Society for Human Genetics and H3Africa Consortium in Senegal, May 2016, and was attended by over 80 participants. The workshop was used to plan and initiate the Kern's six-step approach for designing medical education curricula, which guided development of the AGMT nurse curriculum. Steps included conducting a general needs assessment, followed by specific needs of targeted learners, defining goals and objectives, determining the educational strategies, planning the implementation, and developing an evaluation plan (Kern and Thomas, 2009). The specific needs of targeted learners and defining goals and objectives of the training was guided by the competency mapping strategy from the ISCB (Mulder et al., 2018). Key results from each step are outlined below:

### Step 1: General Needs Assessment

During the AGMT establishment workshop, general needs for new approaches which promote community-based genomic medicine training in Africa were solicited and deliberated by the members present. Data and information generated from this workshop provided the foundation of a survey which was conducted online and advertised through various mailing lists. The survey was conducted to solicit gaps and needs in genomic medicine training from a broad representation of 33 stakeholders and from 19 African countries (https://training.h3abionet.org/AGMC_2016/wp-content/uploads/2017/01/TrainingSurveyAfrica-Upload.pdf). In addition, monthly planning meetings were held to refine the training strategy, develop the curriculum and map competencies, and plan and implement the pilot.

**Figure 1:**
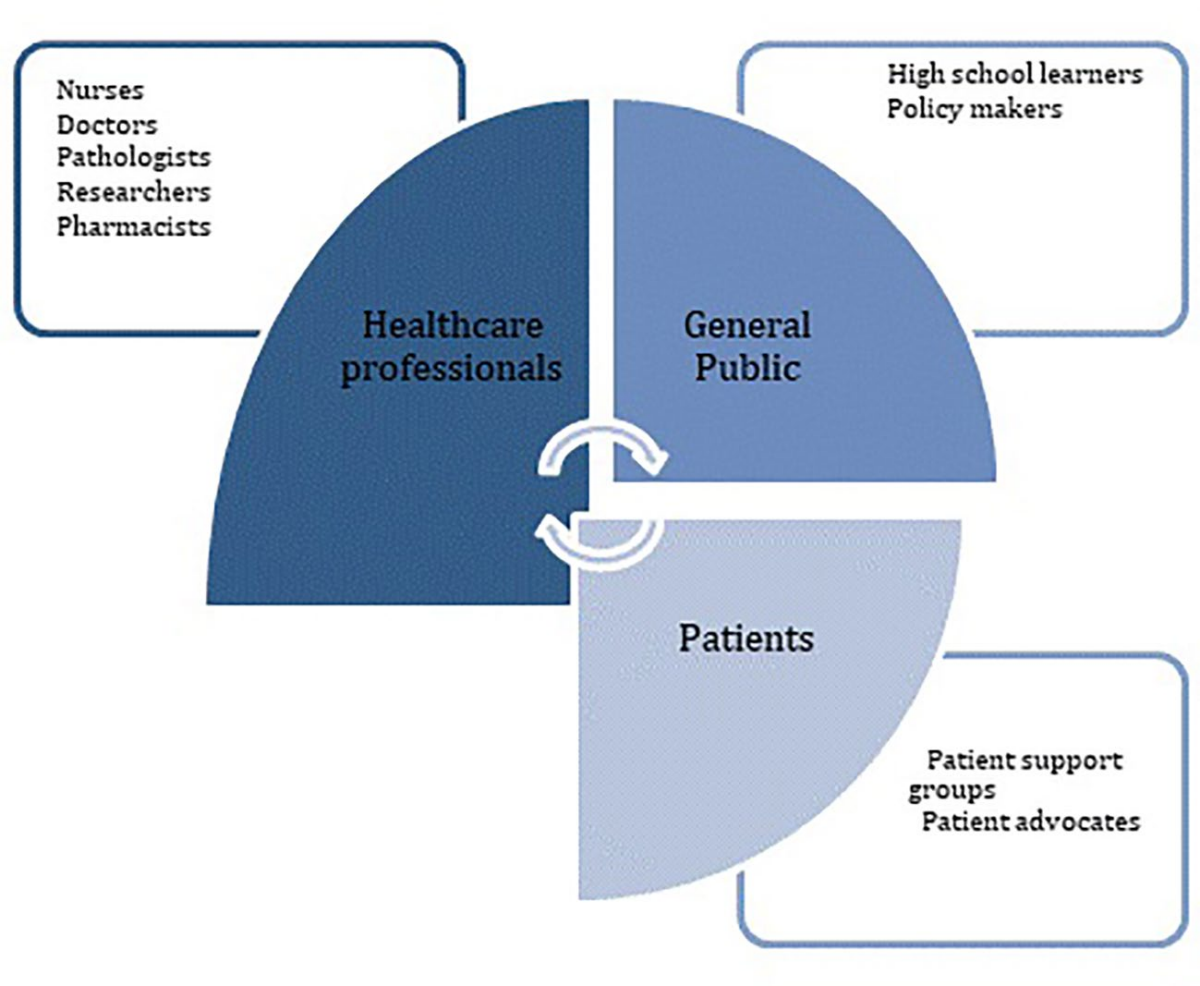
Illustration of key target trainees in genomic medicine for AGMT in the near future. Healthcare workers, patients, and the general public.

From this multiapproach general needs assessment, several gaps were identified which included: limited and not up-to-date curriculum content, lack of expertise in training in genomic medicine relevant fields such as genomics and genetics; and lack of training resources at the various institutes and limited funding. To address these challenges and gaps, the strategy included short courses, which may be developed further into diploma level content, graduate and postgraduate programs for healthcare and research professionals. Training would also target patients, especially those who plan on being advocates in the genetics and genomics fields. Public engagement activities could also be implemented to align with the training developed for healthcare professionals and patients. **Figure 1** illustrates some of the key trainees AGMT could target in the near future. In addition to healthcare workers, it is important to engage/train patients and the general public in genomic medicine. A website (https://training.h3abionet.org/AGMC_2016/) and mailing list were created to facilitate seamless communication. This was made possible through support from the H3Africa Consortium's H3ABioNet, a pan-African bioinformatics network which focuses on genomics capacity development (http://h3abionet.org).

### Step 2: Needs Assessment of Targeted Learners

This step aims to embed the specific training needs of targeted learners and identify specific stakeholders for the curriculum development, implementation, and evaluation. AGMT engaged with the nurse professors/lecturers, recent graduates, and the Global Research Nurses forum to identify specific needs of the nursing community. An article was published on the Global Health Nurses portal (https://globalresearchnurses.tghn.org/articles/preparing-genomic-medicine-nurse-training-africa/) which summarized existing nurse training and highlighted the role of nurses in Africa. In a similar fashion to the ISCB strategy developed for a Bioinformatics curriculum, four nurse personas were created to make explicit the nurses' current roles and their training needs and targeted outcomes in genomic medicine (**Table 1**). Nurses at different professional levels developed the initial personas and their roles based on the common nurse specializations in Africa. We had a professor in nursing who has worked and trained nurses from several different African countries. The team agreed to work with four personas as they were a convenient number that was perceived to be sufficient to capture common nurse roles in different contexts across the continent. Although the effectiveness of the four personas in informing the curriculum might need to be probed the future, it is believed that convenient sampling used, that is small in scale and purposively selected on the basis of crucial criteria, was deemed appropriate by the team and allowed us to focus on our purpose (Punch, 2005). Based on roles of nurses highlighted by the nurse personas and feedback from nurses it became clear that the course needed to emphasize practical application of content into students' current settings and roles using problem-based learning with clinical case studies relevant to Africa. This strategy was critical to highlight the relevance of the course to current clinical practice and increase uptake. In addition, skills in genetic counseling, community engagement, ethical conduct in research, inclusion of genetics and genomics in patient care, and development of health promotion material which included relevant genetics/genomic material were also found to be required. There was also a need to address stigmas and misconceptions of genetics and genomics commonly found in African communities.

**Table 1:** Personas used to create and map competencies.

**Getrude – Research Nurse at the University of Malawi**
Getrude is 29 years old. She holds a Diploma in Nursing and has 5 years’ experience. She is registered with the Malawi Nursing Council. She was recently recruited as a research nurse in a clinical study being conducted at the Malawi Medical College of Medicine and Nursing. Her current duties include:
• Recruiting volunteers for a clinical and genetics research project. The volunteers will be recruited from the Yao tribe.
• Engaging with some villages from the Yao tribe
• Administering of informed consent
• Piloting and implementing the Case Reporting Forms in collaboration with the Study Coordinator
• Overseeing translation of the Informed Consent Forms and the Case Reporting Forms into the Chiyao language
• Taking blood specimens from children and making sure these are stored as per the Standard Operating Procedures
• Record keeping and other administrative duties as per SOPS provided.
• Reports to the Study Coordinator
• Referring study participants to the local clinic for treatment
**Melody – Senior Midwife HIV Specialist: Malawi**
Melody is 40 years old and holds a Senior Nurse Position in a district hospital. She holds a Bachelor degree in Nursing with two postgraduate diplomas in midwifery and HIV & AIDS Care. She is registered with the South African Nursing Council (SANC). She manages the day-to-day nursing operations within the midwifery department. She has 8 years experience in nursing. Her role covers the areas below:
• Maternal health;
• Reproductive health (including genetic counselling);
• Neonatal/child health (including genetic counselling)
*Duties include:*
Coordination of patient care.
Patient consultation, counselling and recommendation of treatment plans this includes the following clients:
• adherence counselling to avoid drug resistant strains of HIV, TB and other infectious agents
• pregnant women
• those with severe drug responses
• Providing consultation and advice to other nurses as a specialist practitioner
• Individual and team supervision.
• Ensure adherence of the unit to hospital and government policies and guidelines as they relate to nursing procedures, standards and practices, administrative and budgetary management.
• Working in collaboration with other healthcare professionals when they are available.
**Douglas - Community Health Nurse: Nigeria**
• Douglas is a 42-year-old Community Health Nurse who holds a 4 year Diploma in Nursing with 10 years’ experience. He works at a clinic in a farming community in Nigeria providing nursing care, health counselling, screening and education to individuals, families and groups in the community with a focus on health promotion.
• *Duties Include*Providing nursing care and preventative health services in community settings and community-based health care facilities.
• Identifying health care needs, priorities and problems of individuals, families and communities.
• Referring individuals or families in need of specialized care or hospitalization
• Coordinating health care interventions at community level.
• Coordinating the care of patients in community settings in consultation with other health professionals and members of health teams.
• Detects high risk factors amongst community members, developing and implementing care plans for the biological, social, and psychological treatment of patients in collaboration with other health professionals.
• Planning and providing personal care, treatments and therapies including administering medications, and monitoring responses to treatment or care plan.
• Planning and participating in health education programmes, health promotion and nurse education activities in clinical and community settings.
• Providing information about prevention of ill-health, treatment and care.
• Supervising and coordinating the work of other nursing, health and personal care workers
**Erensia - General Nurse, Stellenbosch, South Africa**
She holds a Bachelor degree in Nursing. She is registered with the South African Nursing Council as a Nurse (general, community, psychiatry) and midwife. She is currently working in an adult medical ward.
*Duties include:*
• Conducts individualized patient assessment, prioritizing data collection based on the adult or elderly patient’s immediate condition or needs within time frame specified by governing policies, procedures or protocols.
• Develops individualized plans of care patients reflecting on collaborations with other members of the healthcare team.
• Performs appropriate treatments as ordered by physicians in an accurate and timely manner.
• Performs therapeutic nursing interventions as established by individualized plan of care for the adult or elderly patient and his/her family, taking into account the patient’s family history.
• Provides individualized patient/family education customized to the adult or elderly patient and his/her family.
• Documents patient assessment findings, physical/psychosocial responses to nursing intervention and progress towards problem resolution.
• Initiates emergency resuscitative measures according to adult resuscitation protocols.
• Maintains confidentiality in matters related to patient, family and healthcare staff.
• Provides care in a non-judgmental, non-discriminatory manner that is sensitive to the adult or elderly patient’s and family’s diversity, preserving their autonomy, dignity and rights.
• Reports patient condition to the multidisciplinary team during each shift.
• Maintains current competency in General Nursing
• Keeps up to date with current research evidence in order to change policies and procedures to improve healthcare outcomes

### Step 3: Goals and Specific Objectives for the Training Course

In general, the course aims to support improved genetics and genomics knowledge, attitudes and skills for: research nurses in the biomedical field or those aspiring to be research nurses; specialist nurses working in the genomics/genetics field and general nurse practitioners in their day to day duties, or recent graduates. The overall objectives for the nurse personas were to develop and implement a plan of care for patients that incorporates genetic and genomics knowledge and is sensitive to individual and cultural preferences, practices and norms by offering basic genetic counseling to patients and families, and conducting genomics research that is ethical and appropriate to the nurses' context.

Competencies were adapted from the ISCC competency portal (https://genomicseducation.net/competency) to suit the needs of the African continent and these were mapped to the nurse personas in one face to face workshop, Google documents, and several online meetings. The mapping of ISCC to the AGMT nurse competencies were not retained due to numerous rounds of editing. The AGMT competency mapping team was split into three groups to review the personas and map competencies. Two personas were reviewed by two groups only instead of three. Consensus was agreed during monthly meetings, after face to face discussions and *via* Google docs. Once the targeted competencies had been established, the Bloom's taxonomy was used to determine the most appropriate level for a specific nurse by several competency mapping teams (see **Table 2**) for each persona. The final recommended competency to target is indicated in the last column in **Table 2** and was arrived upon after the three competency mapping teams (each team's competency level mapping is colour coded in **Table 2**) had reached a consensus.

**Table 2:** Suggested nurse competencies mapped to nurse personas.

Competencies	No.	Competencies	Melody	Douglas	Getrude	Erensia	Recommended Competency to target
Professional responsibility	1	Ability to engage in reflective practice about one’s own beliefs and values related to patient care that integrates genetics and genomics.	3, 3, 3	1,1	3,2,3	2,2	**3**
	2	Articulate one’s roles and boundaries of one's own professional practice in relation to genetics/genomics.	3, 3,3	3,3	3,1, 3	3,3	**3**
	3	Knowledgeable about relationships which exist between human and/or pathogen genetics, genomics and the environment.	1, 2,1	2,2	3,2, 3	2,2	**2**
	4	Seek coordination and collaboration with an interdisciplinary team of health professionals.	3, 3,3	3,3	3,1, 3	3,3	**3**
Patient Assessment and Care	5	Know and express the difference between clinical diagnosis of disease and identification of genetic predisposition to disease (genetic variation is not strictly correlated with disease manifestation).	3,2,2	1,1	3,2,3	2,2	**2**
	6	Ability to keep up to date with new research evidence in order to understand the importance of Genetics in viral and bacterial infections and treatment regimes.	2,3,2	0,0	2,0,2	1,2	**2**
	7	Demonstrate ability to collect personal, medical and family history that includes genetic/genomic as well as environmental risks.	3,3,3	3,3	3,2,3	3,3	**3**
	8	Ability to incorporate into the inter-professional plan of care the need for further genetic/genomic evaluation or other risk management interventions in collaboration with the client.	3,2,3	2,2	2,1,2	2,2	**2**
	9a	Develop health promotion/disease prevention material that considers genetic and genomic information.	2,2,2	2,2	2,3,2	2,2	**2**
	9b	Apply health promotion/disease prevention practices that consider genetic and genomic information.	2,2,2	2,2	2,3,2	2,2	**2**
	10	Use ethical principles when deliberating genetic/genomic issues of decision making, privacy, confidentiality, informed consent, disclosure, access and personal impact.	3,3,3	3,3	3,3,3	3,3	**3**
	11	Demonstrate use of language and genetic counselling skills appropriate to the client's level of understanding and developmental age when explaining genetic and genomic information.	3,2,3	2,2	3,3,3	2,2	**3**
	12	Ability to integrate best evidence, clinical judgement, client preferences, and family implications in planning genetic and genomic focused individualised care.	3,2,1	2,1	3,1,2	2,3	**2**
Research and Development	13	Identify and continually update resources available to assist clients seeking genetic and genomic information or services including the types of services available.	2,2,2	2,2	2,1,2	2,2	**2**
	14	Demonstrate the ability to use a research protocol and the workflow.	1,1,1	0,0	3,3,3	2,1	**3**
	15	Demonstrate ability to effectively use information technology to obtain credible, current information about genetics and genomics.	2,3,2	1,1	3,2,3	2,2	**3**
	16	Ability to implement quality assurance procedures within a research protocol.	2,3,2	0,0	3,3,3	2,1	**3**
	17a	Understand how to identify disease-associated genetic variations.	1,3,1	0,0	2,2,3	0,2	**2**
	17b	Understand how disease-associated genetic variations facilitate the development of prevention, diagnosis and treatment options.	3,3,3	2,1	3,3,3	2,2	**3**
	19	Ability to develop and implement a community engagement plan for a genetics/genomics research study.	1,3,2	0,2	3,3,3	1,1	**3**

Key: No competency – 0; Awareness – 1: Bloom (Knowlegde and Comprehension); Working knowledge – 2: Bloom (Application and Analysis); Specialist knowledge – 3;Bloom (Synthesis and Evaluation). There are multiple competence levels shown as between 2 and 3 different groups mapped the competencies. The last column in bold was the final competence level selected.

### Step 4: Educational Strategies

This step involves planning the content to be taught and the educational methods to be used. Content was mainly adapted from a genomic medicine curriculum developed by Health Education England to upskill United Kingdom's National Health Service healthcare professionals, in readiness for the implementation of genomic approaches through the 100k Genomes project (https://www.genomicseducation.hee.nhs.uk/; https://www.genomicsengland.co.uk/about-genomics-england/the-100000-genomes-project/). Assessments were adapted to align to the specific competencies identified in step 3. **Table 3** provides a brief description of the final course modules, full details are available on the AGMT website (https://training.h3abionet.org/AGMC_2016/). The four modules varied in length depending on the number of classes they had. Each class was allocated 1 week with contact sessions which lasted around 2 h. Student centered approaches that encourages integration of prior and current experiences were deemed most suitable to facilitate learning for working adults. By selectively drawing on elements of problem-based and project-based learning this enabled the use of real-life questions, a challenge or problem as educational strategies to facilitate the development of knowledge (Lennon et al., 2019). Therefore, several case studies relevant to African health were sourced from the various working group members and embedded in the course material and class assessments. These types of teaching methods are often used for training of health-care professionals as they get students to engage with self-directed learning and offer opportunities for facilitation by the instructor (Kaufman and Holmes, 1996; Kaufman and Holmes 1998).

**Table 3:** Summary of modules included in the training.

Module	Description of module	Lessons
Introductory Module	This module introduces the learners to Genomic Medicine. Provides an overview of key areas in African genomics, human genetics and genetic variation. The history of Genomic Medicine, its relevance to Africa and implications to the nursing profession	Lesson 1 – Overview of Course Lesson 2: Patterns of Genetic Inheritance Lesson 3: Genes, Genome Structure and Function Lesson 4: Molecular Diagnostics and Bioinformatics Techniques
Ethical, Legal and Social Issues	This module introduces participants to ethical, legal and social issues in genomic medicine and research. Principles of community engagement were introduced. More importantly, the learners were taught basic genetic counselling	Lesson 5 – Ethical, Legal and Social Issues in Applied Genomics Lesson 6 – Community Engagement Lesson 7 – Basic Genetic Counselling Skills
Clinical Application of Genetics and Genomics	This module introduces participants to practical examples and case studies in the Genomic Medicine field. The trainers are African based and focus on African-centric examples. In this module participants use their newly acquired basic genetic counselling skills in class and in the clinic.	Lesson 8 – Monogenic Disorders Lesson 9 – Molecular Pathology of Cancer and Application in Cancer Diagnosis, Screening and Treatment Lesson 10 – Application of Genomics to Non-communicable Diseases Lesson 11 – Panel Discussions (Nutrigenomics & Microbiomes) Lecture 12 – Pharmacogenetics & Pharmacogenomics for Nurses in Africa
Research and Genetic Epidemiology	This module introduces participants to research concepts and gives them an opportunity to work on a collaborative research study – if good enough, the study is published	Lesson 13 – Clinical Research and Genetic Epidemiology Lesson 14 – Introduction to Class Mini-Projects

Learning activities such as quizzes, online discussions on the University of Cape Town's learning management system Vula (powered by Sakai), preclass exercises, and postclass assignments were developed in alignment with indicative content and competencies for each lesson. In addition, the range of learning activities was structured to enable students to actively engage and apply their knowledge and thus promote student-centered learning and flipped class learning (Goh and Ong, 2019). Furthermore, exercises and assessments were made relevant to the participants' context as it required participants to produce resources such as generate a list of genetics and genomics resources and services available at their institutes to make it easier for them to refer their patients.

Classes were required to submit a collaborative research project at the end of the course. which aimed to promote collaborative development of publishable research and reviews, and the assignments could be submitted to a special collection in a specific journal. However, classes were also free to choose not to publish their work or publish in a separate journal. The initial plan was for the course to run over six months, however the formatting of class projects into manuscripts continued after the course had concluded and this exercise varied across the different classes.

### Step 5: Implementation

Each classroom is managed by a facilitator who ensures the lectures are played, the class is linked up to the live sessions, and facilitates the interactive activities. We had a brief three-week training for facilitators in three areas; online facilitation; face to face group facilitation; and facilitation of role plays required for genetic counseling. Trainers were asked to develop slides based on the indicative content provided and the aligned competencies and assigned levels. This was a negotiated process.

**Figure 2:**
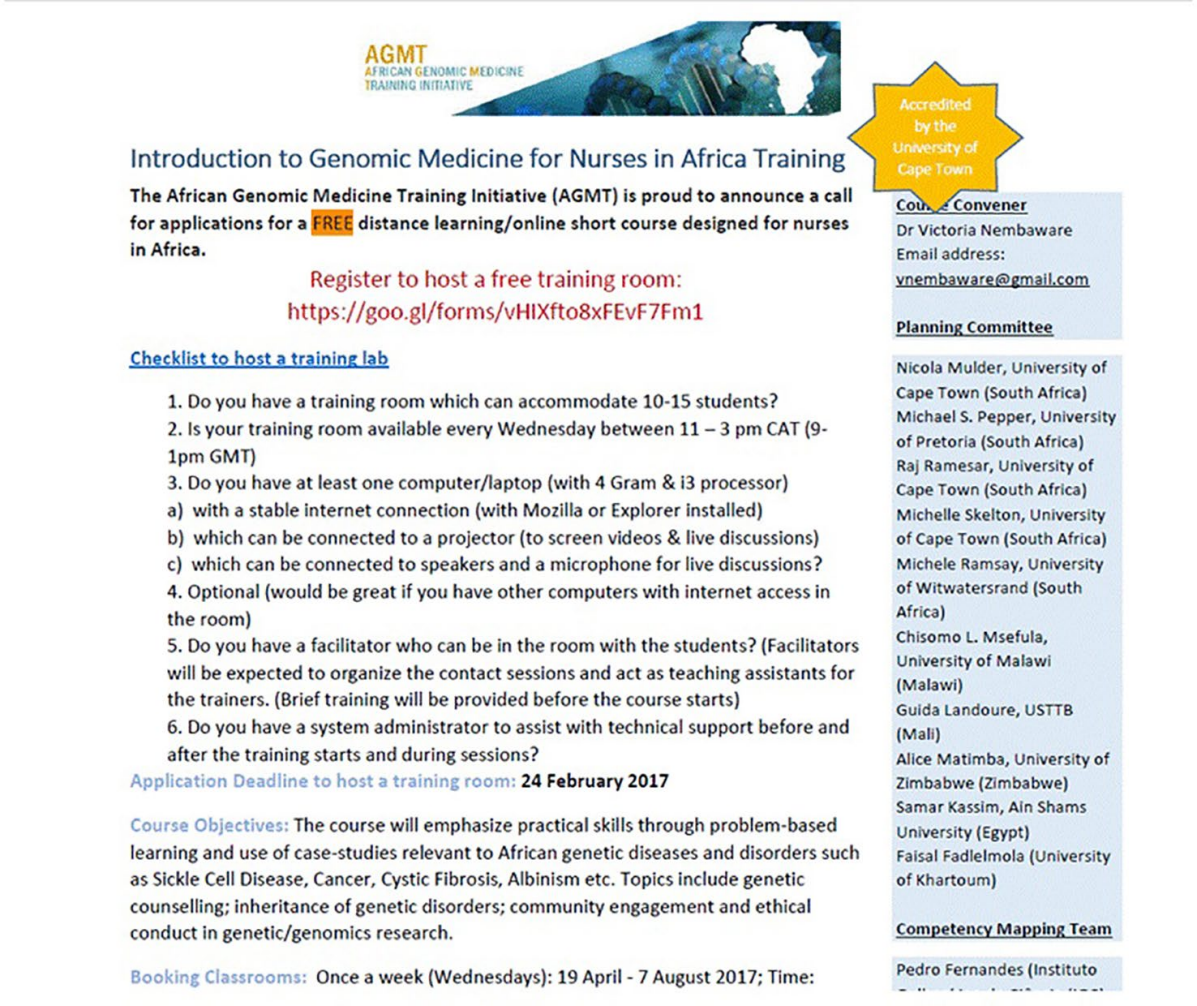
Flier circulated widely across various mailing list to attract the first cohort of trainees.

An electronic advertisement was circulated *via* several mailing lists to advertise the course, see **Figure 2**. Almost 30 different sites applied to host a classroom for the course, however 19 classes were chosen based on their meeting the requirements to provide stable internet connectivity, a qualified facilitator in genetics and hardware that could handle hosting webinars. The coordinator advertised the training extensively *via* social media, the chosen class facilitators were also required to recruit participants in their areas/regions. The course was open to qualified and practising African-based nurses and it ran from April to August in 2017 with a weekly contact session every Wednesday.

**Figure 3:**
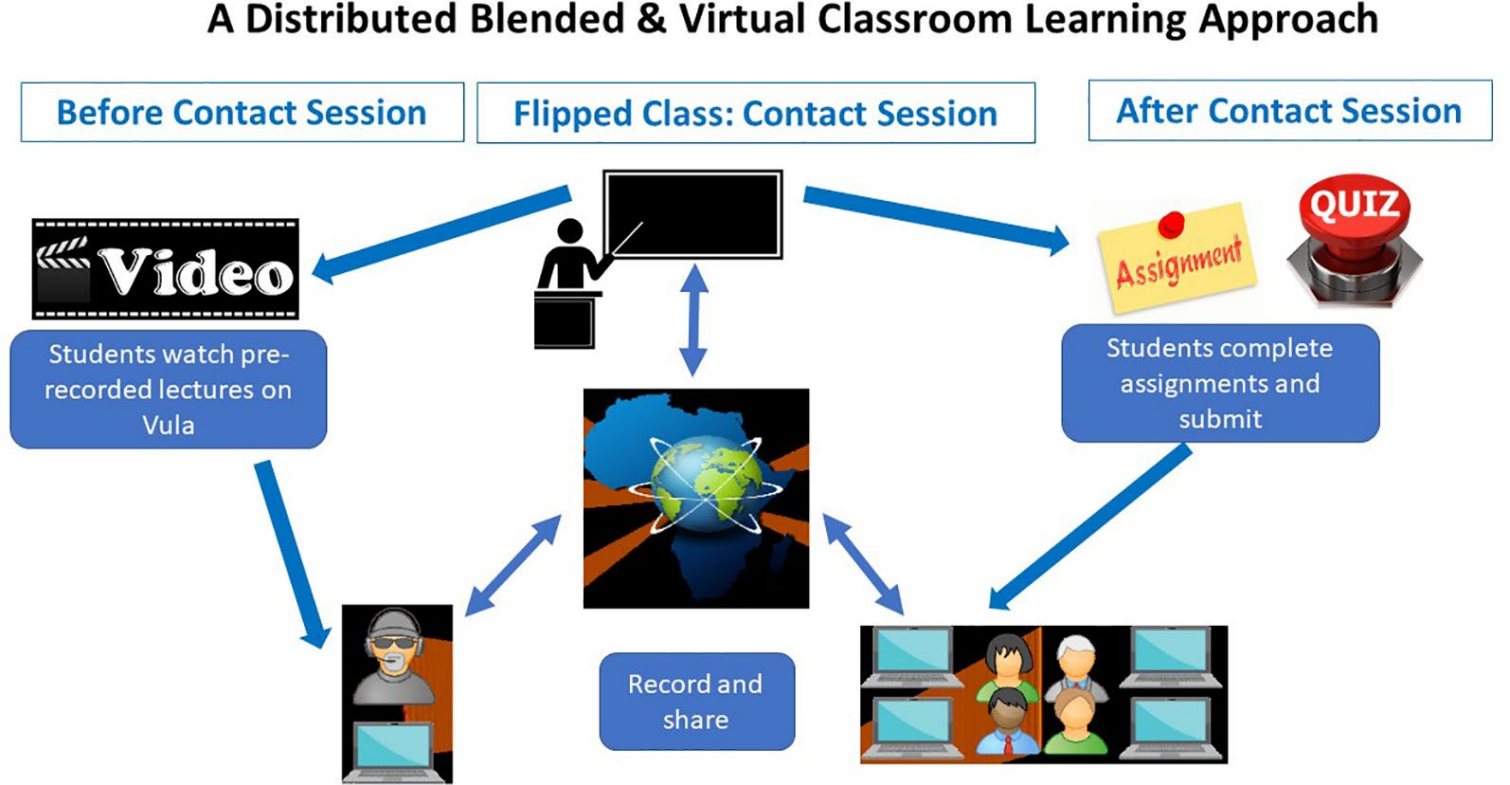
A summary of how the training is conducted using the distributed virtual classroom and blended learning approach. At least 7 days before the face to face sessions, participants can download videos from Vula and watch before class and do preclass exercises. During class (face to face sessions), participants engage with learning material (videos, lectures, etc) during face to face classrooms predownloaded by a facilitator. The class can then connect with the trainer virtually for a question and answer session. Participants also submit a quiz and rate the class. After class participants submit an assignment.

A distributed blended online classroom approach, similar in structure to the Structured Training for African Researchers (STARS) Career development course which was developed through the Association of Commonwealth Universities (https://www.acu.ac.uk/focus-areas/early-careers/structured-training-for-african-researchers/) and was recently adopted and adapted by the H3ABioNet Introduction to Bioinformatics Training course (Gurwitz et al., 2017), was used for the training. For the virtual classroom approach, trainers were required to prerecord their material, which was then loaded onto a learning management system, in this case – Vula (Sakai based). Facilitators of the various classes were then required to download the course material onto local storage devices such as hard drives to avoid relying on internet connectivity during the live weekly contact sessions. The facilitators and learners watched the videos within the physical classrooms distributed across Africa before the 1-h long online contact session with the trainers. The online live sessions were made possible *via* a webinar platform. During these live sessions, only one connection was allowed from each class *via* the facilitator. The class could then pose questions *via* the facilitators. **Figure 3** summarizes the distributed classroom approach.

All facilitators were encouraged to obtain accreditation from relevant bodies across Africa for continued professional development points (CPD). While the initial plan was to obtain accreditation from all affiliated universities for this course as a short course, this was not feasible and therefore the short course accreditation was only obtained from the University of Cape Town. During the first implementation of the project, facilitators were also tasked with marking the qualitative and face to face assessments such as role plays.

### Step 6: Evaluations and Assessments

Two types of assessments were designed for the participants, namely summative and formative analysis (Taras, 2005). Formative assessments included the prelesson exercises which were posted on the Vula discussion forum for all to comment, assess, and give feedback. These prelesson exercises aimed to facilitate the students to acquire key skills or understand concepts that the lesson was targeting to address as required for flipped classes (Riddell et al., 2017). Facilitators also used these preclass exercises to gauge general understanding of the concepts by the participants. In addition, feedback forms were sent to participants after each lesson for the participants to rate content, trainers, and logistics. Classes were also required to submit their research proposals for review by the coordinator and the AGMT working group after lessons on research proposal development. AGMT working group members volunteered to assist/review class proposals that aligned with their own research interests. The summative assessment included the participants completing a knowledge, attitude, perceptions, and practices survey during registration to gauge knowledge at baseline. The same survey was administered at the end of the course and will be administered again 24 months after completion of the course. The summative assessment also included assignments and quizzes given to participants after each lesson. Once the research proposals had been approved by the AGMT working group members, as part of the summative assessment each class was required to write a research report which made up 30% of the students' final marks.

In addition to the participant focused assessments, the overall course's implementation process was also evaluated in order to:

- Understand factors influencing motivation of nurses to sign up for a genomic medicine training course.
- Investigate implementation fidelity, challenges and successes experienced by the facilitators (as described in Step 5).
- Monitor attendance registers and statistics for access to the Vula platform.

The data collection surveys were adapted from literature and used to collect data for each of the assessment strategies highlighted above (McCann et al., 2007; Muzoriana et al., 2017). The participant's assessment strategy and overall implementation evaluation of the project was submitted for review by the Ethics Approval Committee University of Cape Town, Faculty of Health Sciences Ethics Review board.

## Perspective

To facilitate a rapid and informed adoption of genomic medicine into routine clinical care in Africa, Continuous Professional Development (CPD) training and formal higher education for healthcare professionals requires radical transformation suitable for low resource settings. Lack of or inappropriate training could delay the translation of the emerging information from several capacity building efforts in genomics and genetics into quality healthcare (Wonkam and Mayosi, 2014; Weitzel et al., 2016). However, instead of being deterred by the challenges and gaps rampant in Africa, the AGMT course was created to pool resources and expertise across the continent and beyond to provide training for healthcare professionals in genomic medicine which would not have been possible through a single institute or initiative.

While a more thorough evaluation of this program is currently ongoing, preliminary results suggest that this is a feasible model. During the first iteration of the course, 368 applications were received, and 225 participants enrolled into the course from 19 Classrooms in 11 Countries. 35% of the participants completed the course and obtained a certificate of completion of the short course from the University of Cape Town. A special collection was set up by the AGMT to which classes submitted their class projects as manuscripts in this peer-reviewed journal http://gheg-journal.co.uk/2018/05/advancing-genomic-medicine-globally/. So far, one, one class published their class project the special collection. The second iteration of the course is still ongoing.

Nurses are frontline workers in most healthcare facilities in Africa and have access to in-depth knowledge of the patients, families, and communities (Prows et al., 2005). There has been an ongoing debate on whether genetic counselling should only be done by professionally trained genetic counselors or if nurses can receive extra training to enable them to provide basic genetic counseling as a component of their current role (Barr et al., 2018). Based on recent review of 10 articles, Barr et al. (2018) confirmed that nurses already provide genetic counseling, as highlighted by the nurse personas development in this study. However, the provision of genetic counseling by nurses is not standardized. There are calls for formal recognition of the nurses' counseling role and the provision of training to support this task. Because of the lack of genetic counselors on the continent (Abacan et al., 2019) and limited resources to train and employ genetic counselors widely in addition to low job creation for genetic counselors low (Kromberg et al., 2013), providing training in genomics and genetics to nurses who can provide basic genetic counseling seems like a feasible strategy to increase the availability of genetic counseling in Africa.

The importance of establishing a set of core competencies to guide the development of skills, knowledge and attitudes required to deliver safe and effective healthcare is well established (Korf et al., 2014). Competencies in genomics and genetics for nurses have been developed and are publicly available online. However, the alignment of existing curricula to such competencies remains limited or is probably reported poorly. The slow rate of curricula modifications in Europe and America has been largely attributed to lack of implementing personnel and difficulties in operationalization of long and complicated competency lists (Jenkins and Calzone, 2007). Noteworthy, is that developing continents including Africa have been largely underrepresented in such competency development initiatives or curricula development initiatives (Jenkins and Calzone, 2007; Korf et al., 2014). This may be partly due to outdated and static curricula which make the alignment with competencies very difficult because it cannot respond appropriately to societal challenges and needs (Gonzalo et al., 2017).

The draft curriculum and competency map provided from this work are likely to promote increased adoption and adaptation of the genomic medicine training into existing nursing curricula across nurse training colleges and centers across Africa. The embedding of online/distance learning modules into formal university/college training has been demonstrated for various massive online open courses (MOOCs). Although MOOCS were originally developed as stand-alone training to be accessed by university students outside of regular curriculum (Swinnerton et al., 2017), when embedded in university medical curriculum, participants have reported high satisfaction on MOOC sourced-course material (Aboshady et al., 2015). Guidelines would need to be developed to facilitate the inclusion of the AGMT modules into existing university curriculum as done by de Jong et al. (de Jong et al., 2019) for the MOOCs.

Another immediate goal of AGMT is to design training for other healthcare workers such as doctors, pharmacists, clinical scientists, patients, and the general public (e.g., patient support groups). The pilot training program and experiences of the process provides a foundation for the group to develop a toolkit for designing and implementing training for other healthcare workers and possibly offering tailored modules across different professions to reflect the multidisciplinary approach in healthcare systems. Unlike other continents/countries such as Europe (Paneque et al., 2016), Australia (McEwen et al., 2013), and Canada (Ferrier et al., 2013) where bodies have been established to standardize the genetic counseling competencies, where genetic counselors and genetic nurses can register/be certified, most countries in Africa do not yet have certification or registration systems or guidelines to advise on training and practice standards for genomic medicine. The AGMT initiative provides a unique opportunity to be a springboard for development by partnering with existing professional bodies, and by extending training activities to other healthcare professionals.

To our knowledge, this is the first large-scale community-based training initiative for genomic medicine that has been conducted across Africa. This study highlights the importance of societies and consortia in developing a rigorous training program and a pool of trainers and resources for emerging areas such as genomic medicine.

## Data Availability Statement

All datasets generated for this study are included in the article/supplementary material.

## Ethics Statement

Approval was not required according to the study format and local legislation. The paper reports on the implementation of a training program and does not contain personal information.

## Author Contributions

VN and NM developed the first manuscript draft. AGMT members who edited or contributed to the manuscript are listed in alphabetical order in the Appendix. Their role in the project is noted in the first column. Members of the planning team (Kuda Majada, Minnet Cotzee, Faisal Fadlelmola, Pedro Fernandes, Samar Kamal Kassim, Cordelia Leisegang, Ebony Madden, Alice Matimba, Oyekanmi Nash, Michael Pepper, Fouzia Radouani, Raj Ramesar, Michelle Skelton) were responsible for the course development and organization.

## Funding

NM is supported by the National Human Genome Research Institute (NHGRI) and the Office of The Director (OD), the National Institutes of Health under award numbers U41HG006941 and U24HG006941. VN is supported by NIH/NHLBI U24HL135600. The content is solely the responsibility of the authors and does not necessarily represent the official views of the National Institutes of Health. The funders had no role in study design, data collection, and analysis, decision to publish, or preparation of the manuscript.

## Conflict of Interest

The authors declare that the research was conducted in the absence of any commercial or financial relationships that could be construed as a potential conflict of interest.

## Appendix Author Details -Members of the African Genomic Medicine Training Initiative

**Table:**

**Working Group Members**	**Name and**	**Surname**	**Qualifications**	**Expertise**	**Email address**	**Affiliation**
Facilitator	Omar	Abidi	PhD Human genetics	Human genetics & molecular biology Education and Research; Coordinator of Master in Pedagogy of Nursing Sciences and Health Techniques	abidi.or@gmail.com	Institut Supérieur des professions Infirmières et Techniques de Santé (ISPITS) de Casablanca, Morocco
Facilitator	MB	Akanle	B.ENG, MICT	BigData, Machine learning, Cloud Computing, Bioinformatics and Data communication	bola.akanle@covenantuniversity.edu.ng	Covenant University Bioinformatics Research (CUBRe), Nigeria
WG Member, Planner, Advisor	Stuart Alvaro	Ali	PhD, FIBMS, FRSPH	Genetic testing, epidemiology,	stuart.ali@wits.ac.za	Precision Medicine for Africa - A Division of the Wits Health Consortium, University of the Witwatersrand Johannesburg, Republic of South Africa
Facilitator	Cliff Asher	Aliga	MSc Emerg. & Crit Nurse, BSN, PhD Candidate	Emergency and Critical Care Nurse Specialist	cliff.aliga@gmail.com	Aga Khan UNiversity, Uganda
Coordinator	Paballo	Chauke	Msc Biodiversity, Conservation and Managenent BSocSc(Hons)Environemental and Geographical Science	Bioinformatics Training and Outreach Coordinator Bioinformatics education and curriculum development, Bioinformatics Climate Change, Biodiversity Conservation, Social Research, Facilitation and Lecturing	paballo.chauke@uct.ac.za	H3ABioNet, University of Cape Town
Planning Team	Minnet	Cotzee		Professor in Nursing	minette.coetzee@uct.ac.za	Child Nurse Practice Development Initiative, Department of Paediatrics and Child Health, Faculty of Health Sciences, University of Cape Town, Cape Town, South Africa.
Trainer, WG member	Collet	Dandara	BSc, BSc(Hons), PhD	Pharmacogenomics, Drug metabolism, Human Genetics, Genomics, epigenetics and tissue culture	collet.dandara@uct.ac.za	Pharmacogenomics & Drug Metabolism Research Group, Division of Human Genetics, Department of Pathology & Institute of Infectious Disease and Molecular Medicine, University of Cape Town
Planning Team, Facilitator, Trainer	Faisal M.	Fadlelmola	PhD (Human Molecular Genetics & Bioinformatics)	Molecular Biology, Genetics, Genomics, Bioinformatics and Information Technology	faisal.mohamed@hotmail.com	Centre for Bioinformatics & Systems Biology, Faculty of Science, University of Khartoum, P.o. Box 321 Khartoum, Sudan
Facilitator	Michael B.	Fawale	MBBS, MSc Pharmacology, FMCP (neurology)	Clinical Neurology, Stroke genetics, Epilepsy Epidemiology of sleep disorders	bimbofawale@live.com	Obafemi Awolowo University, Ile-Ife, Nigeria
Planning Team, Advisor	Pedro L.	Fernandes		BioinformaticsTraining	pfern@igc.gulbenkian.pt	Instituto Gulbenkian de Ciência, Oeiras, Portugal
Trainer, WG Member	Anita	Ghansah	PhD Genetic Epidemiology	Molecular Biology, Genetics Molecular/genomic epidemiology	aghansah2013@gmail.com	Noguchi Memorial Institute for Medical Research, University of Ghana, Legon-Ghana
Facilitator	Karen	Kengne Kamga	MD	Physician, genetic counselling	kkarenke@yahoo.co.uk	Department of pathology, division of human genetics, University of Capetown
Facilitator	Zainab Abimbola	Kashim	B.Sc., M.Sc. (Microbiology) M.Sc. (Environmental Science	Microbiology, Bioinformatics	bimski4real@gmail.com	National Biotechnology Development Agency, Abuja, Nigeria
Planning Team,Trainer, Lecturer	Samar Kamal	Kassim	MD, PhD Medical Biochemistry and Molecular Biology, Diploma Clinical Nutrition	Medical Biochemistry, Molecular Biology, Genomics, Bioinformatics Education, Clinical Nutrition	samar_kassim@med.asu.ed.eg	Faculty of Medicine, Ain Shams University, Cairo, Egypt
Facilitator	Morenikeji A.	Komolafe	MBBS, Dip. Clin Neurology, FWACP	Genetics, Epilepsy, Parkinson’s Disease, Sleep Disorders.	adeyoyin2001@yahoo.com	Obafemi Awolowo University, Ile-Ife, Nigeria
Trainer	Guida	Landouré	MD, PhD	Human genetics, Neurogenetics	guida@icermali.org	Université des Sciences, des Techniques et des Technologies de Bamako, Mali
Planning Team	Cordelia	Leisegang	B.Nurs., M.Nurs. (Nursing)	Clinical trials, online learning/training and leadership	cordelia.leisegang@outlook.com	Global Research Nurses University of Cape Town/Private
Planning team, WG member,Advisor	Ebony B	Madden	B.S. Biology, M.S. Genetic Counseling, Ph.D. Genetics and Human Genetics	Genetic epidemiology, genomic medicine, population genetics, health disparities	ebony.madden@nih.gov	Division of Genomic Medicine, National Human Genome Research Institute, National Institutes of Health
Planning Team	Kuda	Majada		General Nurse	kudabango@yahoo.com	The School of Nursing, University of Western Cape,
Coordinator	Malebo	Malope	BSc. Medical Sciences, MSc (Med) Genetic Counselling	Genetic Counsellor, Serious Congenital Abnormalities and Termination of Pregnancy	mlpmal005@myuct.ac.za	Department of Pathology, Division of Human Genetics, University of Cape Town, South Africa
Trainer/ WG	Mamadou	Kaba	MD, PhD	Microbiomics; Infectious Diseases; Medical Microbiology; Medical Virology	mamadou.kaba@hotmail.com	Division of Medical Microbiology, Department of Pathology, Faculty of Health Sciences, University of Cape Town, Cape Town, South Africa. Institute of Infectious Disease and Molecular Medicine, University of Cape Town, Cape Town, South Africa
Facilitator	Keofentse	Mathuba		Registered Nurse/Midwife	kmathuba@baylorbotswana.org.bw	Botswana-Baylor Children's Clinical Center of Excellence, Gaborone, Botswana
Trainer, Planning Team	Alice	Matimba		Pharmacogenomics, Training	alice.matimba@wellcomegenomecampus.ac.uk	Advanced Courses and Scientific Conferences, Wellcome Genome Campus, Hunxton, UK
Facilitator	George	Mochamah	MSc. Clinical trials	Clinical trials	gmochamah@kemri-wellcome.org	KEMRI-wellcome Trust, Kilifi
Advisor	Sarah L	Morgan	BSc(Hons), MSc, MA, PhD (Tumour Diagnostics)	Programme and curriculum development (both training and higher education), trainer development, competency assessment, Molecular Biology, Ethics	sarahm@ebi.ac.uk	Training team, EMBL-European Bioinformatics Institute, Hinxton, UK
Planning Team, Trainer	Nicola	Mulder	PhD Medical Microbiology	Bioinformatics education and curriculum development, Bioinformatics, Genomics	nicola.mulder@uct.ac.za	Computational Biology Division, Department of Integrative Biomedical Sciences, IDM, University of Cape Town, Cape Town, South Africa
Planning Team, WG Member	Nash	Oyekanmi		Genomics, Bioinformatics	oyekan.nash@gmail.com	Centre for Genomics Research and Innovation, National Biotechnology Development Agency, Abuja, Nigeria
Trainer, WG member	Nchangwi S	Munung	M.Sc Med M.Sc Biochemistry	Bioethics	nchangwisyntia@yahoo.com	Division of Human Genetics and Institute of Infectious Diseases and Molecular Medicine, Faculty of Health Sciences, University of Cape Town, Cape Town, South Africa
Coordinator, Trainer	Victoria	Nembaware		Bioinformatics, Genomics, Training, Capacity Building	vnembaware@gmail.com	Division of Human Genetics and Institute of Infectious Diseases and Molecular Medicine, Faculty of Health Sciences, University of Cape Town, Cape Town, South Africa
Facilitator	Temitope Esther	Owoeye		Molecular Biology, Genetics and Genomics	topmost0504@yahoo.com	National Biotechnology Development Agency, South-West Centre, Ibadan, Nigeria
Planning Team, Working Group, Trainer	Michael Sean	Pepper	MBChB, PhD, MD, PD	Genomics, cell and gene therapy	michael.pepper@up.ac.za	Institute for Cellular and Molecular Medicine, Faculty of Health Sciences, University of Pretoria
Advisor	Lunelle	Pienaar		Staff development and curriculum development	lunelle.pienaar@uct.ac.za	Department of Health Sciences Education UCT
Trainer	Elize	Pietersen	PhD (Med)	Clinical health research	elize.pietersen@gmail.com	University of Cape Town / Private
Planning Team, WG member, Facilitator	Fouzia	Radouani	PhD (Immunology	PhD Immunology; Research in Infectious diseases & public health problems; Molecular biology; Genomics and genetics; Bioinformatics (tools & database development, data analysis)	radouani@gmail.com	Chlamydiae and Mycoplasmas Laboratory, Institut Pasteur du Maroc, Morocco
Planning Team, Working Group Member	Raj	S Ramesar		Human Genetics	raj.ramesar@uct.ac.za	MRC Genomic and Precision Medicine Research Unit, Division of Human Genetics, University of Cape Town
Planning Team, WG Member	Michelle	Skelton		Human Genetics	michelle.skelton@uct.ac.za	H3Africa Administrative Coordinating Centre University Of Cape Town, South Africa
WG member, Facilitator	Sununguko Wata	Mpoloka		Molecular Biology, Population Genetics	mpoloka@ub.ac.bw	Department of Biological Sciences, University of Botswana (and CAfGEN)
WG Member	Wayengerera	Misaki	MD, PhD	Human Genetics & Genomics	waymisaki@gmail.com	Department of Medical Microbiology, College of Health Sciences, Makerere University, Kampala, Uganda
WG Member	Ambroise	Wonkam	MD, PhD	Genetic medicine Human Genetics Medical Ethics	ambroise.wonkam@uct.ac.za	Division of Human Genetics and Institute of Infectious Diseases and Molecular Medicine, Faculty of Health Sciences, University of Cape Town, Cape Town, South Africa
Facilitator, Trainer	Adetunji Samuel	Adesina	MSc (Biochemistry and Molecular Biology)	Molecular Biology and Bioinformatics	asadesina@yahoo.com	Department of Biochemistry and Molecular Biology, Obafemi Awolowo University, Ile-Ife, Nigeria.
Trainer	Tina-Marié	Wessels	MSc(Med) Genetic Counselling (PhD)	Genetic Counsellor Training	tina.wessels@uct.ac.za	Division Human Genetic Genetics, Department of Pathology, University of Cape Town
